# Measurement properties of the Dutch PROMIS-29 v2.1 profile in people with and without chronic conditions

**DOI:** 10.1007/s11136-022-03171-6

**Published:** 2022-06-25

**Authors:** Ellen B. M. Elsman, Leo D. Roorda, Nynke Smidt, Henrica C. W. de Vet, Caroline B. Terwee

**Affiliations:** 1 Department of Epidemiology and Data Science, Amsterdam Public Health Research Institute, Amsterdam UMC, Vrije Universiteit Amsterdam, de Boelelaan 1089a, 1081 HV Amsterdam, The Netherlands; 2 Amsterdam Rehabilitation Research Center | Reade, Amsterdam, The Netherlands; 3 Department of Epidemiology, University Medical Center Groningen, University of Groningen, Groningen, The Netherlands

**Keywords:** PROMIS, Psychometric properties, Patient-reported outcomes, Validation, Mental health, Physical health

## Abstract

**Purpose:**

To investigate the structural validity, internal consistency, measurement invariance, and construct validity of the Dutch PROMIS-29 v2.1 profile, including seven physical (e.g., pain, physical function), mental (e.g., depression, anxiety), and social (e.g., role functioning) domains of health, in a Dutch general population sample including subsamples with and without chronic diseases.

**Methods:**

The PROMIS-29 was completed by 63,602 participants from the Lifelines cohort study. Structural validity of the PROMIS-29, including unidimensionality of each domain and the physical and mental health summary scores, was evaluated using factor analyses (criteria: CFI ≥ 0.95, TLI ≥ 0.95, RMSEA ≤ 0.06, SRMR ≤ 0.08). Internal consistency, measurement invariance (no differential item functioning (DIF) for age, gender, administration mode, educational level, ethnicity, chronic diseases), and construct validity (hypotheses on known-groups validity and correlations between domains) were assessed per domain.

**Results:**

The factor structure of the seven domains was supported (CFI = 0.994, TLI = 0.993, RMSEA = 0.046, SRMR = 0.031) as was unidimensionality of each domain, both in the entire sample and the subsamples. Model fit of the physical and mental health summary scores reached the criteria, and scoring coefficients were obtained. Cronbach’s alpha for the seven PROMIS-29 domains ranged from 0.75 to 0.96 in the complete sample. No DIF was detected. Of the predefined hypotheses, 78% could be confirmed.

**Conclusion:**

Sufficient structural validity, internal consistency and measurement invariance were found, both in the entire sample and in subsamples with and without chronic diseases. Requirements for sufficient evidence for construct validity were (almost) met for most subscales. Future studies should investigate test–retest reliability, measurement error, and responsiveness of the PROMIS-29.

**Supplementary Information:**

The online version contains supplementary material available at 10.1007/s11136-022-03171-6.

## Introduction

Patient-reported outcome measures (PROMs) are questionnaires that assess the perspective of patients regarding their health. The patients’ perspectives have become increasingly important for clinical decision making, and in health research and policy making [1–3]. The use of PROMs enables monitoring symptoms and evaluating treatment effectiveness and can enhance communication between patients and clinicians to improve the engagement of patients in their care [4, 5].

The Patient-Reported Outcomes Measurement Information System (PROMIS®) is an initiative founded by a collaboration of eight US research institutes and the US National Institutes of Health. PROMIS aims to standardize the measurement of patient-reported outcomes by developing a standardized set of high-quality PROMs based on modern psychometric techniques (called item banks) to assess core physical (e.g., pain, physical function), mental (e.g., depression, anxiety), and social (e.g., role functioning) domains of health [6–8]. PROMIS item banks can be administered using computerized-adaptive testing (CAT) or through fixed-length and custom-made short forms [9]. In addition, several PROMIS profile instruments are available containing a fixed number of items from seven PROMIS core health domains (physical function, pain interference, anxiety, depression, fatigue, sleep disturbance, and ability to participate in social roles and activities), measured on 5-point Likert scales, plus a 0–10 numeric rating item on pain intensity [10]. With 29 items, the PROMIS-29 v2.1 profile is the shortest profile. It consists of four items for each of the seven domains, equivalent to the standard 4-item short forms, plus the single pain intensity item [11]. The PROMIS-29 is more or less comparable to the Short-Form 36 Health Survey (SF-36) [12], one of the most widely used profile measures today. However, it measures slightly different domains and was developed based on the results of item response theory (IRT) [13, 14] instead of classical test theory (CTT). The length of the PROMIS-29 is relatively short while providing a wealth of health-related information because each domain is scored separately [11]. Moreover, Hays et al. have developed physical and mental health summary scores [15] analogous to the global physical health and a global mental health scores of the PROMIS Global Health Scale [16] and the physical and mental component scores of the SF-36 [17]. These bottom-line indicators can be of value [18], and allow the PROMIS-29 to be used as other, older instruments.

PROMIS item banks or their short forms have been translated into more than 60 languages, including Dutch [19]. Psychometric assessments of various Dutch item banks have been conducted [20–25], including the assessment of cross-cultural validity (absence of differential item functioning (DIF) for language), making them available for use in the Netherlands in research and clinical practice. Because PROMIS profiles combine short forms on the core domains of health [10], these profiles are particularly suitable for use in clinical trials, observational studies, and routine clinical practice. With PROMIS profiles, a broad overview of a person’s health status can be obtained, which is particularly useful for patients with multiple conditions or comorbidities impacting several health domains.

The applicability of the seven Dutch-Flemish PROMIS item banks on which the PROMIS-29 is based is supported so far by results of IRT analyses, including the absence of DIF for language [20–27]. However, there is no evidence yet for the seven-factor structure of the PROMIS-29 domains in the Netherlands, neither in the general population nor in persons with chronic diseases. It would also be important to know whether the physical and mental health summary score and the associated factor scoring coefficients of Hays et al. [15] can be reproduced in another sample. Moreover, for most item banks, [28] included in the PROMIS-29 measurement invariance for persons with and without chronic diseases as well as for other important sociodemographic characteristics (e.g., ethnicity, educational level), has not been assessed. Therefore, the objective of this study was to investigate the structural validity of the PROMIS-29, including unidimensionality of each domain and its physical and mental health summary scores. Moreover, internal consistency, measurement invariance (no DIF for age, gender, mode of administration, educational level, ethnicity, and chronic diseases), and construct validity (hypotheses on known-groups validity and correlations between domains) were assessed for each domain of the PROMIS-29.

## Methods

### Participants

For this cross-sectional study, data were obtained from the Lifelines cohort study. Lifelines are a multi-disciplinary prospective population-based cohort study examining the health and health-related behaviors of 167,729 persons living in the North of the Netherlands in a unique three-generation design. It employs a broad range of investigative procedures in assessing the biomedical, sociodemographic, behavioral, physical, and psychological factors which contribute to the health and disease of the general population, with a special focus on multi-morbidity and complex genetics [29]. The study population is broadly representative for the people living in this region [30]. Detailed information about the cohort and participant selection can be found elsewhere [29, 31, 32]. Before participating in the cohort all participants provided written informed consent. The Lifelines cohort study is approved by the medical ethics committee of the University Medical Center Groningen, the Netherlands. The Lifelines cohort study is conducted in accordance with the ethical standards as laid down in the Declaration of Helsinki. For the present study, adults of 18 years and older who completed the PROMIS-29 v2.1 profile were included. The PROMIS-29 was administered in Lifelines follow-up 2B during the period 2016–2020, for which 109,407 adults were invited.

### Measures

Participants completed questions regarding their demographic characteristics (age, gender, educational level, and ethnicity) and the presence of chronic diseases (diabetes, cardiovascular disease, chronic obstructive pulmonary disease (COPD), high blood pressure, and other chronic diseases). Participants also completed the Dutch version of the PROMIS-29 v2.1 profile [19]. The PROMIS-29 v2.1 profile contains the standard 4-item short forms from seven PROMIS core health domains (physical function, pain interference, anxiety, depression, fatigue, sleep disturbance, and ability to participate in social roles and activities) and one separate item on pain intensity from the PROMIS Global Health scale. Each item has 5 response options, except for the pain intensity item, which has a 0–10 numeric rating scale. All items have a seven-day recall period, except for the items in the domains ‘physical function’ and ‘ability to participate in social roles and activities’, for which the recall period is not indicated [11] (PROMIS measures can be obtained through healthmeasures.net). Total scores for each domain are derived from the IRT model and expressed as T-scores with a mean of 50 and a standard deviation of 10 for the US reference population [33]. Higher T-scores indicate a higher level of the underlying construct. Because of the large sample size it was not possible to calculate T-scores by uploading item scores in the online HealthMeasures Scoring Service, provided by the US Assessment Center [34]. Therefore, T-scores were calculated by obtaining the official US item parameters used in the US Assessment Center through enquiry.

### Statistical analyses

**Fig. 1:**
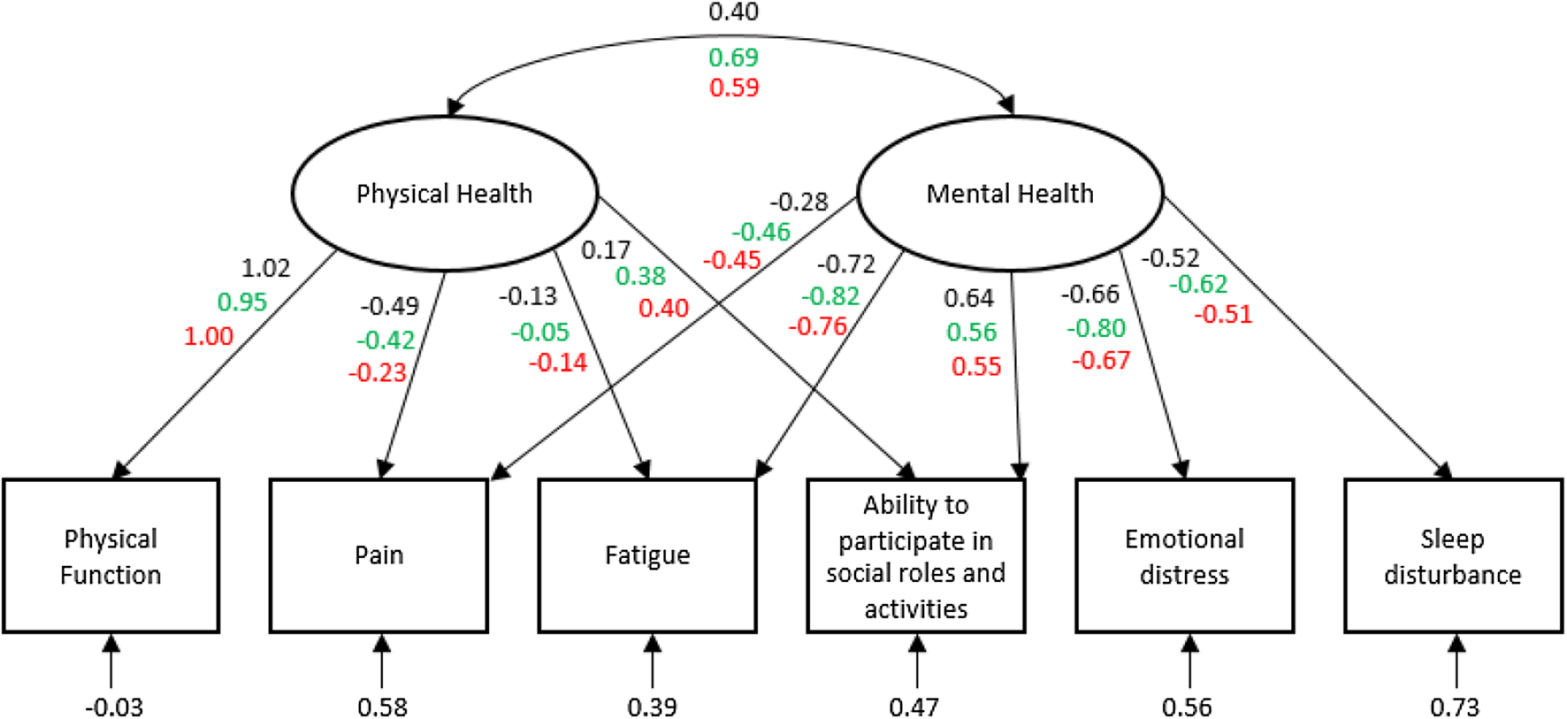
Standardized CFA estimates for the physical and mental health summary scores. Numbers above the squares represent standardized factor loadings, numbers below the squares represent standardized error variances; Black: standardized factor loadings from this study, green: standardized factor loadings from the study of Hays et al. [15], red: standardized factor loadings from the study of Huang et al. [35]; Pain is average of pain interference and pain intensity item, emotional distress is average of anxiety and depression. (Color figure online)

All analyses were conducted in R-Studio or SPSS version 25. Descriptive statistics were used to analyze demographic and clinical characteristics of participants and the percentage of participants with the minimum or maximum score. Structural validity was investigated with confirmatory factor analyses (CFA) in the R package lavaan [35]. First, a seven-factor correlated CFA was fitted, examining the expected factor structure of the PROMIS-29 as a whole, both for the entire sample and separately for participants with and without chronic diseases. Next, items from each domain separately were fitted to a single-factor CFA in order to assess the unidimensionality of each short form. This was also done for the entire sample and for participants with and without chronic diseases. Because of the ordinal response options diagonally weighted least squares (DWLS) estimation with a mean- and variance-adjusted test statistic (weighted least square mean and variance (WLSMV)) was used. Last, a two-factor correlated CFA with maximum likelihood estimation was fitted with domain z-scores to investigate the structural validity of the physical and mental health summary scores. As advised by Hays [15, 36], a pain composite was created by averaging z-scores for the pain intensity item and the pain interference domain to minimize local dependence. In addition, an emotional distress composite was created by averaging z-scores for depressive symptoms and anxiety domains. Similar to the model of Hays et al. [15], the factor physical health was represented by z-scores for physical function, pain (composite score), fatigue, and ability to participate in social roles and activities. The factor mental health was represented by z-scores for fatigue, pain (composite score), ability to participate in social roles and activities, emotional distress (composite score), and sleep disturbance (see also Fig. 1). For all models, CFA model fit was evaluated using the following criteria [37]: Comparative Fit Index (CFI) ≥ 0.95, Tucker-Lewis Index (TLI) ≥ 0.95, root mean square error of approximation (RMSEA) ≤ 0.06, and standardized root mean square residual (SRMR) ≤ 0.08. Standardized factor loadings were compared to the loadings reported by Hays et al. [15] and Huang et al. [38]. Subsequently, factor scoring coefficients for the physical and mental health summary scores were estimated with linear regression models in which the factor scores were the dependent variable and the z-scores for each of the domains were the independent variables.

To evaluate internal consistency, Cronbach’s alpha was calculated for each of the seven PROMIS-29 domains for the entire sample and for participants with and without chronic diseases. To assess measurement invariance, DIF analyses for each domain were conducted with an iterative hybrid of logistic regression and IRT with the R package lordif [39]. The likelihood-ratio *χ*^2^ test with detection criterion *R*2 was used to detect DIF. McFadden’s pseudo-*R*^2^ was used as a measure of DIF magnitude with a 2% change being considered as critical threshold. DIF was assessed for age (median split: ≤ 53 years and ≥ 54 years), gender, mode of administration (digital vs. paper and pencil), educational level (high vs. medium/low), ethnicity (Dutch nationality vs. other), and chronic diseases (no vs. yes, and each of the chronic diseases vs. no chronic disease). No DIF was expected for any of these variables given the intended universal applicability of the PROMIS-29 [40]. With respect to construct validity, known-group validity was assessed for groups that were expected to differ in score: groups differing in age (three age groups were compared), gender, and chronic diseases (yes/no) were evaluated. The expected direction and magnitude of the differences were based on previous research on other Dutch adults on the same domains [22, 25–27, 41]. Furthermore, Pearson correlations between each of the domains and the pain intensity item were calculated. The magnitude and direction of the expected correlation was based on previous knowledge on and experience with the measured constructs. In total, 88 a priori hypotheses were formulated (see Table 6). In line with the COSMIN (COnsensus-based Standards for the selection of health Measurement INstruments) methodology [42] if at least 75% of the hypotheses were confirmed the construct validity of the PROMIS-29 was considered sufficient.

## Results

A total of 63,602 respondents completed the PROMIS-29 (response rate 58%). Those who completed the PROMIS-29 had a higher mean age at baseline (47.8 vs. 42.4 years), were more often female (58.8% vs. 57.2%), more often had a low educational level at baseline (31.9% vs. 26.2%), and were more often native Dutch (94.9% vs. 94.0%). Table 1 presents the characteristics of the respondents. For each item, all response categories were endorsed. Missing responses on each of the items ranged from 0.2 to 1.3%. Depending on the direction of scoring of the domain, the number of respondents having minimum or maximum raw sum score (i.e., the best score) was high, especially for physical function, depression, and pain interference (Table 2).

**Table 1:** Sociodemographic characteristics of participants

Sociodemographic characteristic	Complete sample^a^ (*n* = 63,602)	Sample without any chronic diseases^a^ (*n* = 39,146)	Sample with chronic disease(s)^a^ (*n* = 24,456)
Age in years, mean ± SD (range)	53 ± 13 (21–96)	51 ± 12 (21–95)	58 ± 12 (21–96)
18–39	14.6	19.5	6.7
40–64	64.0	66.5	60.0
≥ 65	21.4	14.0	33.3
Gender			
Male	40.8	41.6	39.5
Female	59.2	58.4	60.5
Mode of administration			
Digital	66.7	70.8	60.1
Paper and pencil	33.3	29.2	39.9
Educational level			
Low	31.1	25.7	39.8
Middle	35.4	36.6	33.4
High	33.5	37.7	26.8
Ethnicity			
Native	95.0	95.0	94.8
1st and 2nd generation western immigrant	4.0	3.9	4.1
1st and 2nd generation non-western immigrant	1.1	1.1	1.1
Chronic disease			
No	61.5	100	0.0
Cardiovascular disease	6.4	0.0	16.5
Diabetes	3.6	0.0	9.5
COPD	3.6	0.0	9.3
High blood pressure	13.5	0.0	35.0
Other	20.5	0.0	53.3

*COPD* chronic obstructive pulmonary disease, *SD* standard deviation

^a^All results expressed as % unless otherwise noted

**Table 2:** PROMIS-29 mean T-scores per domain, and the percentage participants having the maximum and minimum score, for the complete sample and samples with and without chronic diseases

	Physical function	Ability to participate in social roles and activities	Anxiety	Depression	Fatigue	Sleep disturbance	Pain interference	Pain intensity^a^
Complete sample (*n* = 63,602)								
Mean T-score (SD)	52.9 (6.7)	55.1 (8.0)	48.0 (8.2)	45.9 (7.2)	45.3 (9.4)	46.8 (7.0)	47.7 (8.1)	1.8 (2.3)
Maximum score %	68.5	34.1	0.0	0.0	0.5	0.2	0.4	0.1
Minimum score %	0.1	0.3	46.3	64.6	28.4	3.4	59.7	43.0
Without chronic diseases (*n* = 39,146)								
Mean T-score (SD)	54.7 (5.0)	56.2 (7.5)	47.4 (7.9)	45.3 (6.8)	44.0 (8.8)	46.1 (6.6)	45.8 (6.8)	1.4 (1.9)
Maximum score %	79.6	38.2	0.0	0.0	0.2	0.1	0.1	0.0
Minimum score %	0.1	0.1	48.9	67.9	32.1	3.8	69.3	51.1
With chronic diseases (*n* = 24,456)								
Mean T-score (SD)	50.0 (8.0)	53.4 (8.5)	49.0 (8.6)	46.8 (7.8)	47.4 (10.0)	47.9 (7.3)	50.6 (9.0)	2.6 (2.5)
Cardiovascular disease (*n* = 4,043)	48.6 (8.5)	53.4 (8.6)	48.5 (8.3)	46.4 (7.5)	47.6 (9.7)	47.4 (7.3)	50.6 (9.2)	2.6 (2.6)
Diabetes (*n* = 2,319)	48.7 (8.8)	53.7 (8.8)	48.3 (8.5)	46.9 (7.8)	47.2 (10.3)	47.7 (7.5)	51.2 (9.5)	2.8 (2.6)
COPD (*n* = 2,279)	48.1 (8.7)	52.7 (8.8)	49.0 (8.8)	47.1 (8.1)	48.4 (10.2)	48.1 (7.6)	50.9 (9.3)	2.7 (2.6)
High blood pressure (*n* = 8,570)								
Other (*n* = 13,043)	50.5 (7.9)	54.5 (8.2)	48.5 (8.4)	48.5 (8.4)	45.9 (9.6)	47.5 (7.1)	49.9 (8.8)	2.4 (2.5)
	49.0 (8.3)	51.9 (8.7)	49.9 (8.8)	46.2 (7.4) 47.7 (8.2)	49.4 (10.2)	48.7 (7.4)	52.3 (9.3)	3.1 (2.6)
Maximum score %	50.6	27.4	0.0	0.1	0.9	0.3	0.8	0.1
Minimum score %	0.3	0.5	42.0	59.1	22.5	2.9	44.3	29.8

T-scores: higher scores represent more of the underlying construct; please note that these are not Dutch reference scores, as the sample was not representative for the general Dutch population

*COPD* chronic obstructive pulmonary disease, *SD* standard deviation

^a^Pain intensity is not a T-score but a 0–10 numeric rating scale

Satisfactory CFA model fit was found for the entire PROMIS-29, confirming its seven-factor structure both for the complete sample as for the samples with and without chronic diseases (Table 3). The model provides acceptable fit to the response data. Each single-factor CFA for each domain separately also had acceptable model fit in all three samples, although the cut-off for RMSEA was not met for all domains. The measurement model, thus, seems to make conceptual sense for the assessments of the domains and the items included in the domains [43]. Factor loadings for the seven-factor model and each single-factor model can be found in Supplementary file 1.

**Table 3:** CFA model fit for the entire PROMIS-29 and all domains tested separately, and Cronbach’s alpha

	PROMIS-29	Physical function	Ability to participate in social roles and activities	Anxiety	Depression	Fatigue	Sleep disturbance	Pain interference
Complete sample (*n* = 63,602)								
CFI scaled (> 0.95)	0.994	0.998	0.998	0.992	0.999	0.999	0.994	0.999
TLI scaled (> 0.95)	0.993	0.995	0.994	0.977	0.997	0.997	0.984	0.999
RMSEA scaled (< 0.06)	0.046	0.077*	0.115*	0.188*	0.080*	0.165*	0.112*	0.099*
SRMR (< 0.08)	0.031	0.016	0.012	0.032	0.010	0.017	0.031	0.006
Cronbach’s alpha		0.89	0.91	0.89	0.91	0.94	0.75	0.96
Without chronic diseases (*n* = 39,146)								
CFI scaled (> 0.95)	0.993	0.997	0.998	0.991	0.999	0.999	0.995	0.999
TLI scaled (> 0.95)	0.992	0.992	0.994	0.974	0.996	0.996	0.984	0.999
RMSEA scaled (< 0.06)	0.040	0.055	0.107*	0.182*	0.076*	0.159*	0.102*	0.087*
SRMR (< 0.08)	0.032	0.021	0.013	0.034	0.011	0.019	0.033	0.007
Cronbach’s alpha		0.82	0.90	0.88	0.90	0.93	0.71	0.95
With chronic diseases (*n* = 24,456)								
CFI scaled (> 0.95)	0.993	0.998	0.998	0.993	0.999	0.999	0.994	0.999
TLI scaled (> 0.95)	0.992	0.993	0.995	0.979	0.997	0.997	0.982	0.999
RMSEA scaled (< 0.06)	0.053	0.104*	0.128*	0.197*	0.086*	0.173*	0.132*	0.113*
SRMR (< 0.08)	0.032	0.019	0.012	0.030	0.010	0.015	0.031	0.006
Cronbach’s alpha		0.89	0.92	0.90	0.91	0.94	0.79	0.97

*Not meeting cut-off criterion

Figure 1 shows the standardized estimates from the CFA of the physical and mental health summary scores with domain z-scores for the total population. Standardized factor loadings were similar to those found by Hays et al. [15] and Huang et al. [38], although the correlation between the two factors was notably lower (*r* = 0.40 in this study vs. *r* = 0.69 and *r* = 0.59 in the studies of Hays et al. [15] and Huang et al. [38], respectively). Model fit reached the criteria: CFI = 0.982, TLI = 0.947, RMSEA = 0.080, SRMR = 0.025. Table 4 shows scoring coefficients to calculate the physical and mental health summary scores.

**Table 4:** Scoring coefficients for the physical and mental health summary scores from the CFA model (scoring coefficients found by Hays et al. [15] in parentheses)

Domain	Physical health summary score	Mental health summary score
Physical function	1.546 (0.872)	− 0.073 (− 0.015)
Pain^a^	0.030 (− 0.094)	− 0.122 (− 0.154)
Ability to participate in social roles and activities	− 0.011 (0.113)	0.361 (0.252)
Fatigue	0.008 (− 0.009)	− 0.417 (− 0.351)
Emotional distress^b^	− 0.002 (0.003)	− 0.362 (− 0.257)
Sleep disturbance	− 0.001 (0.002)	− 0.221 (− 0.139)

Scoring coefficients can be used to calculate physical and mental health summary scores. Taking the Physical Health Summary (PHS) score as an example: PHS_z = (physical function z-score*1.546) + (pain z-score *0.030) + (ability to participate z-score*-0.011) + (fatigue z-score*0.008) + (sleep disturbance z-score*-0.002) + (emotional distress z = score*-0.001); PHS = PHS_Z*10 + 50

^a^Pain is average of pain interference and pain intensity item

^b^Emotional distress is average of anxiety and depression

The estimated physical and mental health summary scores are presented in Table 5, calculated with the scoring coefficients presented in Table 4 and with the scoring coefficients developed by Hays et al. [15]. On a population level, physical and mental health summary scores based on the Dutch scoring coefficients were approximately one T-score point higher than physical and mental health summary scores based on the US scoring coefficients. However, on an individual level, absolute differences between the two scoring approaches reached up to eight points for the mental health summary score and even 20 points for the physical health summary score.

**Table 5:** PROMIS physical and mental health summary T-scores based on Dutch and US scoring coefficients

	Mean population T-score (SD) [range] with Dutch scoring coefficients	Mean population T-score (SD) [range] with US scoring coefficients	Mean absolute difference (SD) [range]
Physical health summary score	54.3 (10.1) [7.2–61.9]	53.3 (6.8) [20.1–58.8]	3.3 (1.8) [0.0–20.6]
Mental health summary score	55.7 (8.9) [13.2–70.1]	54.5 (7.1) [20.6–65.7]	1.9 (1.2) [0.0–8.3]

T-scores, higher scores represent more physical/mental health; please note that these are not Dutch reference scores, as the sample was not representative for the general Dutch population

*SD* standard deviation

Cronbach’s alpha for each of the seven PROMIS-29 domains ranged from 0.75 to 0.96 in the complete sample (Table 3), showing that the domains do not include items beyond their concept [43]. Cronbach’s alpha for each domain was higher in the sample with chronic diseases compared to the sample without chronic diseases.

No DIF for age, gender, mode of administration, educational level, ethnicity, or presence of chronic diseases was detected for any of the domains (McFadden’s pseudo-*R*^2^ all < 0.02; Supplementary file 2). Nor was DIF detected in each of the chronic diseases compared to no chronic disease for any of the domains (McFadden’s pseudo-R^2^ all < 0.02; Supplementary file 3). Differences in demographic backgrounds, thus, do not lead to substantially different interpretations of the items in each of the domains, nor do different modes of administration lead to substantially different scores. Also, the scoring rule does not create bias with respect to one group of patients versus another [43].

Of the predefined hypotheses, 78% could be confirmed (64%-100% per subscale) (Table 6). The hypotheses not being confirmed were mostly related to the one point difference between adjacent age groups in the first hypotheses. The domain sleep disturbance had the least confirmed hypotheses (64%). The large number of confirmed hypotheses shows that scores from most domains correspond to how persons actually feel or function in their daily lives, and that the scores are sensitive enough to reflect differences in the domains between persons [43]. The T-scores of the groups can be found in Supplementary file 4, whereas the Pearson correlations among PROMIS-29 domains and the pain intensity item are presented in Supplementary file 5.

**Table 6:** Confirmation of a priori hypotheses regarding the expected differences between groups and the correlation between domains of the PROMIS-29

Hypotheses	Number confirmed
Older participants score better than younger participants on the domains fatigue, sleep disturbance, anxiety and depression; a difference of at least 1 point is expected between each adjacent age group (18–39, 40–64, ≥ 65)^a^	2/8
Younger participants score better than older participants on the domains physical function and pain interference and on pain intensity; a difference of at least 1 point is expected between each adjacent age group (18–39, 40–64, ≥ 65) for physical function and pain interference, and a difference of at least 0.5 point for pain intensity^a^	5/6
The youngest (18–39) and oldest (≥ 65) age group score at least 1 point better than the middle age group (40–64) on the domains ability to participate in social roles and activities^a^	0/2
Males score at least 1 point better than females on all domains, and 0.5 point better on pain intensity^a^	7/8
Participants without chronic diseases score at least 2 points better than people with chronic diseases on all domains, and 1 point better on pain intensity^a^	5/8
The following domains have a correlation between 0.6 and 0.7: physical function and pain interference (negative), physical function and pain intensity (negative), anxiety and depression, sleep disturbance and fatigue	4/8
The domains ability to participate in social roles and activities and physical function have a correlation between 0.4 and 0.6	2/2
The domain pain interference has a correlation of at least 0.6 with pain intensity	2/2
The remaining domains have a correlation of less than 0.5 (depending on the direction, this might be negative)	42/44
Total	69/88 (78%)
Physical function	10/11 (91%)
Ability to participate in social roles and activities	8/11 (73%)
Anxiety	8/11 (73%)
Depression	8/11 (73%)
Fatigue	9/11 (82%)
Sleep disturbance	7/11 (64%)
Pain interference	11/11 (100%)
Pain intensity	8/11 (73%)

^a^Better means higher T-scores for the domains physical function and ability to participate in social roles and activities, and lower T-scores for the domains anxiety, depression, fatigue, sleep disturbance, pain interference; for pain intensity, better means a lower score on the 0–10 numeric scale

## Discussion

This study assessed some important measurement properties of the Dutch PROMIS-29 in a large cohort. We found sufficient evidence for structural validity, internal consistency, and measurement invariance, both in a sample with and without chronic diseases, whereas requirements for sufficient evidence for construct validity were (almost) met for most subscales. Therefore, the PROMIS-29 is considered a valid instrument to measure physical, mental, and social aspects of self-reported health in adults with and without chronic diseases for use in research and routine clinical practice.

We found a high proportion of participants obtaining the minimum and maximum score (i.e., the best score, depending on the direction of the domain) for most domains, in accordance with findings from previous studies in general population samples [44, 45]. Particularly, over 50% of the population obtained the best scores in the domains physical function, depression, and pain interference. Only the domain sleep disturbance seems to be an exception with only few participants obtaining the minimum score, which is also consistent with other studies [44, 45]. The number of participants with a minimum or maximum score was lower in the sample with chronic diseases. However, even within the sample with chronic diseases, more than 50% of participants had the maximum score for the domain physical function and the minimum score for the domain depression. This latter result was also found in a study with patients with rheumatic diseases [46]. There, thus, seems to be some mistargeting of the short-form items included in the PROMIS-29, even though these items were selected from the item banks following a mix of qualitative expert input and quantitative criteria [10]. Indeed, if we look at the item parameters (obtained from the US Assessment Center in order to calculate T-scores), item parameters for physical function and ability to participate in social roles and activities are all on the lower side of the theta scale. This means that these short forms are more targeted towards persons with low levels of these constructs. For fatigue and sleep disturbance, the item parameters seem to be more equally divided over the theta scale, which possibly also explains the smaller proportion of extreme scores found on these scales. For pain interference, depression, and anxiety the item parameters are on the higher side of the theta scale, and thus, these short forms are more targeted towards persons with high levels of these constructs. The use of CATs has shown to result in a lower proportion of participants obtaining the minimum and maximum score, and CAT scores are accurate over a wider range of the measured construct while only a small number of items is administered [47]. Therefore, to obtain accurate scores with which people are sufficiently discriminated, administration of a CAT might be preferred over these 4-item short forms both in persons with and without chronic diseases.

The seven-factor structure of the PROMIS-29 could be confirmed for the Dutch population and model fit was acceptable for both the entire population as for samples with and without chronic diseases. Unidimensionality for each of the PROMIS domains was also demonstrated. To a certain extent, we were able to reproduce the correlated factor structure for the physical and mental health summary scores. Applying the same model as Hays et al. [15] is in line with PROMIS convention to use the same factor structure for the same measures across the world, unless evidence is provided that this is not acceptable. Since the model fitted quite well and alternative models showed less adequate fit (data not shown), we decided to adhere to this factor structure, which contributes to the general applicability of the scoring system for PROMIS instruments. Although standardized factor loadings were comparable to those found in previous studies [15, 38], the correlation between the physical and mental component was considerably lower. An explanation for this might be that the samples in previous studies were less healthy. The sample of Hays et al. reported about half a standard deviation worse health compared to the general population [15, 48] whereas the sample of Huang et al. consisted of older adults with chronic conditions [38]. Less healthy populations usually have more variations in their responses, resulting in higher correlations. The impact of using the Dutch scoring coefficients versus the US scoring coefficients was small on a population level. Because our sample is broadly representative for the people living in the Northern part of the Netherlands and is over 20 times larger compared to the (less healthy) population from the study of Hays et al. [15, 48], we think our scoring coefficients might be closer to the true values than the scoring coefficients presented by Hays et al. [15]. Therefore, we recommend to use the Dutch scoring coefficients to calculate physical and mental health summary scores for the Dutch population and possibly also for other populations. However, more research is needed to better evaluate this scoring system and replicate the findings, preferably in large (*n* > 50,000) samples like ours.

Cronbach’s alpha values were all around 0.9 or higher, except for sleep disturbance (alpha = 0.75), thereby showing sufficient internal consistency. These results are in accordance with other studies that have also found high Cronbach’s alpha values for PROMIS profile domains [15, 38, 44, 46, 49], with the study of Hays et al. also finding a lower Cronbach’s alpha for sleep disturbance [15].

We assessed DIF for important sociodemographic and clinical characteristics as DIF for language has already been investigated for most full item banks [22–26]. No DIF for age, gender, mode of administration, educational level, ethnicity, or the presence of chronic diseases was detected for any of the domains, nor for any of the chronic diseases separately compared to no chronic disease. The absence of DIF for chronic diseases is of particular importance because the PROMIS-29 is suitable for use in, for example, research or routine clinical practice in which persons with chronic diseases are overly represented.

Of our a priori defined hypotheses 78% could be confirmed, thereby meeting the 75% required for sufficient construct validity according to the COSMIN criteria for good measurement properties [42]. For most domains, this criterion was also (almost) met. Although we based our hypothesis on analyses with other Dutch datasets [22, 25–27, 41] and previous experiences, one should note that a one point difference, as used in some hypotheses, might not (always) be meaningful. It is not yet clear what a minimal important difference in scores between groups is for PROMIS measures, but most studies suggest a within-person change of at least three points to be meaningful [50–54]. However, expecting larger differences between, e.g., age groups would not have been realistic. Another way to formulate hypotheses in future studies is to state that differences smaller than, e.g., 2 points were expected between certain groups. These hypotheses might especially be useful when small, non-meaningful differences are to be expected. Even though the magnitude of the differences between groups was sometimes smaller than expected, especially the differences between adjacent age groups, the direction of the differences was mostly in accordance with expectations. All together, we think our results add to the evidence for sufficient construct validity of the PROMIS-29 domains [15, 46, 49, 55].

A strength of this study is the very large sample size, enabling us to perform the analyses for subgroups with and without chronic diseases and to investigate DIF for important sociodemographic and clinical characteristics. A limitation of our study is the representativeness of the Lifelines cohort, in which males, younger persons, and persons with an immigration background are underrepresented compared with the general Dutch population. Furthermore, in our sample, 62% reported not having a chronic condition, whereas according to registries in 2019, 43% of the Dutch population had no chronic condition [56]. Thus, our sample was not representative for the Dutch population, and therefore, reported T-scores should not be interpreted as reference values for the Dutch population. Papers regarding reference values for the Dutch population on the domains included in the PROMIS-29 have recently been or will soon be published [25, 26, 41]. Finally, formulating challenging hypotheses in which both the direction and the magnitude of the difference or relationship are included, is difficult. We based our hypotheses on findings of previous research, to show that PROMIS-29 functions in our population as expected.

## Conclusion

This study provides evidence for sufficient structural validity, internal consistency, and measurement invariance of the PROMIS-29 profile in the Dutch population. Requirements for evidence for construct validity were (almost) met for most subscales, adding to the evidence for sufficient construct validity. That these measurement properties were sufficient in a sample with chronic diseases and without chronic diseases are important because the PROMIS-29 can be used in, for example, research or routine clinical practice, in which persons with chronic diseases are usually over-represented. The large proportion of participants obtaining the best score on the PROMIS-29 might hamper the ability to discriminate between persons. Therefore, administration of a CAT might be preferred. Future studies should also investigate the test–retest reliability, measurement error, and responsiveness of the PROMIS-29.

## Supplementary Information

Below is the link to the electronic supplementary material.

Supplementary file1 (PDF 276 kb)
