# Supplementary material for: Measurement properties of the Dutch PROMIS-29 v2.1 profile in people with and without chronic conditions
Source: Qual Life Res. 2022 Jun 25;31(12):3447–58. doi: 10.1007/s11136-022-03171-6 (PMC9587921; doi:10.1007/s11136-022-03171-6)
Supplement: Supplementary file 1 — Supplementary file1 (PDF 276 kb) [file 11136_2022_3171_MOESM1_ESM.pdf]

Measurement properties of the Dutch PROMIS-29 v2.1 profile in people with and without chronic conditions

Ellen BM Elsman, Leo D Roorda, Nynke Smidt, Henrica CW de Vet, Caroline B Terwee

Supplementary file 1. Factor loadings for the seven-factor model of the PROMIS-29 and each of the single-factor models, for the complete sample and samples with and without chronic diseases

Item	Factor loadings for the entire PROMIS-29			Factor loadings for each domain tested separately		
	Complete sample (n=63,602)	Without chronic diseases (n=39,146)	With chronic diseases (n=24,456)	Complete sample (n=63,602)	Without chronic diseases (n=39,146)	With chronic diseases (n=24,456)
PFA11	0.937	0.916	0.937	0.896	0.860	0.882
PFA21	0.870	0.826	0.858	0.898	0.863	0.885
PFA23	0.916	0.906	0.892	0.940	0.934	0.927
PFA53	0.698	0.957	0.959	0.961	0.955	0.953
EDANX01	0.872	0.861	0.883	0.893	0.885	0.900
EDANX40	0.908	0.902	0.909	0.933	0.931	0.932
EDANX41	0.910	0.902	0.916	0.883	0.876	0.889
EDANX53	0.914	0.914	0.913	0.893	0.886	0.898
EDDEP04	0.896	0.897	0.894	0.908	0.907	0.906
EDDEP06	0.947	0.945	0.945	0.926	0.928	0.922
EDDEP29	0.915	0.915	0.913	0.919	0.915	0.920
EDDEP41	0.943	0.942	0.942	0.949	0.948	0.948
HI7	0.969	0.965	0.971	0.969	0.965	0.970
AN3	0.969	0.970	0.968	0.977	0.978	0.976
FATEXP41	0.925	0.912	0.931	0.899	0.882	0.908
FATEXP40	0.923	0.911	0.929	0.920	0.908	0.927
Sleep109	0.879	0.889	0.879	0.914	0.915	0.916
Sleep116	0.557	0.515	0.63	0.508	0.470	0.563
Sleep20	0.915	0.902	0.909	0.902	0.895	0.901
Sleep44	0.736	0.707	0.753	0.718	0.691	0.739
SRPPER11_CaPS	0.866	0.856	0.873	0.878	0.862	0.889
SRPPER18_CaPS	0.913	0.902	0.920	0.927	0.918	0.933
SRPPER23_CaPS	0.921	0.899	0.932	0.866	0.848	0.875
SRPPER46_CaPS	0.921	0.911	0.930	0.939	0.929	0.945
PAININ9	0.976	0.971	0.975	0.978	0.972	0.978
PAININ22	0.994	0.993	0.993	0.994	0.994	0.993
PAININ31	0.942	0.924	0.940	0.935	0.917	0.932
PAININ34	0.977	0.972	0.975	0.975	0.971	0.973

Measurement properties of the Dutch PROMIS-29 v2.1 profile in people with and without chronic conditions

Ellen BM Elsman, Leo D Roorda, Nynke Smidt, Henrica CW de Vet, Caroline B Terwee

Supplementary file 2. McFadden's pseudo R^2 values of the PROMIS-29 for sociodemographic characteristics and presence of chronic diseases

Item	McFadden's pseudo R^2					
	**Gender**	**Ethnicity**	**Age**	**Educational level**	**Mode of administration**	**Presence of chronic diseases**
PFA11	R^2_{12} = 0.0018	R^2_{12} = 0.0000	R^2_{12} = 0.0000	R^2_{12} = 0.0001	R^2_{12} = 0.0002	R^2_{12} = 0.0003
	R^2_{23} = 0.0000	R^2_{23} = 0.0000	R^2_{23} = 0.0003	R^2_{23} = 0.0001	R^2_{23} = 0.0000	R^2_{23} = 0.0003
PFA21	R^2_{12} = 0.0000	R^2_{12} = 0.0000	R^2_{12} = 0.0009	R^2_{12} = 0.0003	R^2_{12} = 0.0003	R^2_{12} = 0.0001
	R^2_{23} = 0.0000	R^2_{23} = 0.0000	R^2_{23} = 0.0000	R^2_{23} = 0.0000	R^2_{23} = 0.0000	R^2_{23} = 0.0002
PFA23	R^2_{12} = 0.0034	R^2_{12} = 0.0001	R^2_{12} = 0.0008	R^2_{12} = 0.0002	R^2_{12} = 0.0002	R^2_{12} = 0.0003
	R^2_{23} = 0.0003	R^2_{23} = 0.0000	R^2_{23} = 0.0000	R^2_{23} = 0.0001	R^2_{23} = 0.0001	R^2_{23} = 0.0000
PFA53	R^2_{12} = 0.0002	R^2_{12} = 0.0000	R^2_{12} = 0.0029	R^2_{12} = 0.0005	R^2_{12} = 0.0005	R^2_{12} = 0.0007
	R^2_{23} = 0.0001	R^2_{23} = 0.0000	R^2_{23} = 0.0001	R^2_{23} = 0.0000	R^2_{23} = 0.0000	R^2_{23} = 0.0000
EDANX01	R^2_{12} = 0.0023	R^2_{12} = 0.0000	R^2_{12} = 0.0003	R^2_{12} = 0.0007	R^2_{12} = 0.0013	R^2_{12} = 0.0002
	R^2_{23} = 0.0000	R^2_{23} = 0.0000	R^2_{23} = 0.0000	R^2_{23} = 0.0000	R^2_{23} = 0.0001	R^2_{23} = 0.0001
EDANX40	R^2_{12} = 0.0000	R^2_{12} = 0.0001	R^2_{12} = 0.0006	R^2_{12} = 0.0001	R^2_{12} = 0.0004	R^2_{12} = 0.0000
	R^2_{23} = 0.0000	R^2_{23} = 0.0000	R^2_{23} = 0.0000	R^2_{23} = 0.0000	R^2_{23} = 0.0000	R^2_{23} = 0.0001
EDANX41	R^2_{12} = 0.0003	R^2_{12} = 0.0002	R^2_{12} = 0.0002	R^2_{12} = 0.0007	R^2_{12} = 0.0003	R^2_{12} = 0.0000
	R^2_{23} = 0.0000	R^2_{23} = 0.0000	R^2_{23} = 0.0000	R^2_{23} = 0.0000	R^2_{23} = 0.0000	R^2_{23} = 0.0000
EDANX53	R^2_{12} = 0.0037	R^2_{12} = 0.0000	R^2_{12} = 0.0000	R^2_{12} = 0.0003	R^2_{12} = 0.0000	R^2_{12} = 0.0000
	R^2_{23} = 0.0001	R^2_{23} = 0.0000	R^2_{23} = 0.0000	R^2_{23} = 0.0000	R^2_{23} = 0.0002	R^2_{23} = 0.0000
EDDEP04	R^2_{12} = 0.0009	R^2_{12} = 0.0002	R^2_{12} = 0.0000	R^2_{12} = 0.0000	R^2_{12} = 0.0001	R^2_{12} = 0.0001
	R^2_{23} = 0.0001	R^2_{23} = 0.0000	R^2_{23} = 0.0001	R^2_{23} = 0.0000	R^2_{23} = 0.0001	R^2_{23} = 0.0000
EDDEP06	R^2_{12} = 0.0011	R^2_{12} = 0.0001	R^2_{12} = 0.0002	R^2_{12} = 0.0000	R^2_{12} = 0.0002	R^2_{12} = 0.0005
	R^2_{23} = 0.0001	R^2_{23} = 0.0000	R^2_{23} = 0.0000	R^2_{23} = 0.0000	R^2_{23} = 0.0000	R^2_{23} = 0.0000
EDDEP29	R^2_{12} = 0.0010	R^2_{12} = 0.0000	R^2_{12} = 0.0000	R^2_{12} = 0.0001	R^2_{12} = 0.0002	R^2_{12} = 0.0000
	R^2_{23} = 0.0001	R^2_{23} = 0.0000	R^2_{23} = 0.0000	R^2_{23} = 0.0000	R^2_{23} = 0.0000	R^2_{23} = 0.0000
EDDEP41	R^2_{12} = 0.0009	R^2_{12} = 0.0000	R^2_{12} = 0.0000	R^2_{12} = 0.0000	R^2_{12} = 0.0001	R^2_{12} = 0.0001
	R^2_{23} = 0.0000	R^2_{23} = 0.0000	R^2_{23} = 0.0000	R^2_{23} = 0.0000	R^2_{23} = 0.0000	R^2_{23} = 0.0000
HI7	R^2_{12} = 0.0000	R^2_{12} = 0.0000	R^2_{12} = 0.0007	R^2_{12} = 0.0005	R^2_{12} = 0.0001	R^2_{12} = 0.0000
	R^2_{23} = 0.0000	R^2_{23} = 0.0000	R^2_{23} = 0.0000	R^2_{23} = 0.0000	R^2_{23} = 0.0000	R^2_{23} = 0.0000
AN3	R^2_{12} = 0.0000	R^2_{12} = 0.0000	R^2_{12} = 0.0001	R^2_{12} = 0.0003	R^2_{12} = 0.0001	R^2_{12} = 0.0001
	R^2_{23} = 0.0000	R^2_{23} = 0.0000	R^2_{23} = 0.0002	R^2_{23} = 0.0001	R^2_{23} = 0.0000	R^2_{23} = 0.0004
FATEXP41	R^2_{12} = 0.0003	R^2_{12} = 0.0000	R^2_{12} = 0.0000	R^2_{12} = 0.0009	R^2_{12} = 0.0002	R^2_{12} = 0.0001
	R^2_{23} = 0.0000	R^2_{23} = 0.0000	R^2_{23} = 0.0003	R^2_{23} = 0.0004	R^2_{23} = 0.0000	R^2_{23} = 0.0000

FATEXP40	R^2_{12} = 0.0001	R^2_{12} = 0.0000	R^2_{12} = 0.0010	R^2_{12} = 0.0017	R^2_{12} = 0.0007	R^2_{12} = 0.0001
	R^2_{23} = 0.0001	R^2_{23} = 0.0000	R^2_{23} = 0.0000	R^2_{23} = 0.0000	R^2_{23} = 0.0000	R^2_{23} = 0.0001
Sleep109	R^2_{12} = 0.0001	R^2_{12} = 0.0000	R^2_{12} = 0.0021	R^2_{12} = 0.0006	R^2_{12} = 0.0007	R^2_{12} = 0.0007
	R^2_{23} = 0.0001	R^2_{23} = 0.0000	R^2_{23} = 0.0001	R^2_{23} = 0.0001	R^2_{23} = 0.0000	R^2_{23} = 0.0001
Sleep116	R^2_{12} = 0.0000	R^2_{12} = 0.0001	R^2_{12} = 0.0059	R^2_{12} = 0.0053	R^2_{12} = 0.0000	R^2_{12} = 0.0002
	R^2_{23} = 0.0000	R^2_{23} = 0.0001	R^2_{23} = 0.0000	R^2_{23} = 0.0013	R^2_{23} = 0.0001	R^2_{23} = 0.0000
Sleep20	R^2_{12} = 0.0015	R^2_{12} = 0.0001	R^2_{12} = 0.0038	R^2_{12} = 0.0005	R^2_{12} = 0.0000	R^2_{12} = 0.0005
	R^2_{23} = 0.0000	R^2_{23} = 0.0000	R^2_{23} = 0.0005	R^2_{23} = 0.0000	R^2_{23} = 0.0001	R^2_{23} = 0.0001
Sleep44	R^2_{12} = 0.0047	R^2_{12} = 0.0000	R^2_{12} = 0.0010	R^2_{12} = 0.0006	R^2_{12} = 0.0015	R^2_{12} = 0.0001
	R^2_{23} = 0.0000	R^2_{23} = 0.0000	R^2_{23} = 0.0001	R^2_{23} = 0.0004	R^2_{23} = 0.0002	R^2_{23} = 0.0001
SRPPER11_CaPS	R^2_{12} = 0.0007	R^2_{12} = 0.0000	R^2_{12} = 0.0007	R^2_{12} = 0.0002	R^2_{12} = 0.0000	R^2_{12} = 0.0001
	R^2_{23} = 0.0000	R^2_{23} = 0.0000	R^2_{23} = 0.0000	R^2_{23} = 0.0000	R^2_{23} = 0.0000	R^2_{23} = 0.0000
SRPPER18_CaPS	R^2_{12} = 0.0005	R^2_{12} = 0.0000	R^2_{12} = 0.0002	R^2_{12} = 0.0002	R^2_{12} = 0.0000	R^2_{12} = 0.0001
	R^2_{23} = 0.0001	R^2_{23} = 0.0000	R^2_{23} = 0.0001	R^2_{23} = 0.0000	R^2_{23} = 0.0000	R^2_{23} = 0.0000
SRPPER23_CaPS	R^2_{12} = 0.0025	R^2_{12} = 0.0000	R^2_{12} = 0.0011	R^2_{12} = 0.0000	R^2_{12} = 0.0000	R^2_{12} = 0.0010
	R^2_{23} = 0.0000	R^2_{23} = 0.0000	R^2_{23} = 0.0001	R^2_{23} = 0.0000	R^2_{23} = 0.0001	R^2_{23} = 0.0001
SRPPER46_CaPS	R^2_{12} = 0.0000	R^2_{12} = 0.0000	R^2_{12} = 0.0001	R^2_{12} = 0.0005	R^2_{12} = 0.0001	R^2_{12} = 0.0001
	R^2_{23} = 0.0000	R^2_{23} = 0.0000	R^2_{23} = 0.0000	R^2_{23} = 0.0000	R^2_{23} = 0.0000	R^2_{23} = 0.0001
PAININ9	R^2_{12} = 0.0018	R^2_{12} = 0.0000	R^2_{12} = 0.0005	R^2_{12} = 0.0002	R^2_{12} = 0.0004	R^2_{12} = 0.0002
	R^2_{23} = 0.0000	R^2_{23} = 0.0000	R^2_{23} = 0.0006	R^2_{23} = 0.0001	R^2_{23} = 0.0002	R^2_{23} = 0.0001
PAININ22	R^2_{12} = 0.0001	R^2_{12} = 0.0000	R^2_{12} = 0.0002	R^2_{12} = 0.0000	R^2_{12} = 0.0001	R^2_{12} = 0.0001
	R^2_{23} = 0.0000	R^2_{23} = 0.0000	R^2_{23} = 0.0000	R^2_{23} = 0.0001	R^2_{23} = 0.0000	R^2_{23} = 0.0000
PAININ31	R^2_{12} = 0.0005	R^2_{12} = 0.0000	R^2_{12} = 0.0000	R^2_{12} = 0.0012	R^2_{12} = 0.0003	R^2_{12} = 0.0004
	R^2_{23} = 0.0000	R^2_{23} = 0.0000	R^2_{23} = 0.0000	R^2_{23} = 0.0000	R^2_{23} = 0.0000	R^2_{23} = 0.0000
PAININ34	R^2_{12} = 0.0057	R^2_{12} = 0.0000	R^2_{12} = 0.0000	R^2_{12} = 0.0000	R^2_{12} = 0.0000	R^2_{12} = 0.0001
	R^2_{23} = 0.0000	R^2_{23} = 0.0000	R^2_{23} = 0.0002	R^2_{23} = 0.0000	R^2_{23} = 0.0000	R^2_{23} = 0.0000

Measurement properties of the Dutch PROMIS-29 v2.1 profile in people with and without chronic conditions

Ellen BM Elsman, Leo D Roorda, Nynke Smidt, Henrica CW de Vet, Caroline B Terwee

Supplementary file 3. McFadden's pseudo R^2 values of the PROMIS-29 for chronic diseases

Item	McFadden's pseudo R^2				
	**Cardiovascular disease**	**Diabetes**	**COPD**	**High blood pressure**	**Other**
PFA11	R^2_{12} = 0.0001	R^2_{12} = 0.0002	R^2_{12} = 0.0002	R^2_{12} = 0.0001	R^2_{12} = 0.0022
	R^2_{23} = -0.0127	R^2_{23} = 0.0000	R^2_{23} = -0.0728	R^2_{23} = 0.0001	R^2_{23} = 0.0006
PFA21	R^2_{12} = 0.0013	R^2_{12} = 0.0009	R^2_{12} = 0.0031	R^2_{12} = 0.0008	R^2_{12} = 0.0002
	R^2_{23} = 0.0002	R^2_{23} = -0.0003	R^2_{23} = -0.0673	R^2_{23} = 0.0001	R^2_{23} = 0.0000
PFA23	R^2_{12} = 0.0000	R^2_{12} = 0.0005	R^2_{12} = 0.0001	R^2_{12} = 0.0000	R^2_{12} = 0.0025
	R^2_{23} = 0.0000	R^2_{23} = 0.0002	R^2_{23} = 0.0000	R^2_{23} = 0.0000	R^2_{23} = 0.0003
PFA53	R^2_{12} = 0.0027	R^2_{12} = 0.0026	R^2_{12} = 0.0034	R^2_{12} = 0.0024	R^2_{12} = 0.0001
	R^2_{23} = -0.0010	R^2_{23} = -0.0006	R^2_{23} = -0.0004	R^2_{23} = 0.0000	R^2_{23} = 0.0000
EDANX01	R^2_{12} = 0.0002	R^2_{12} = 0.0001	R^2_{12} = 0.0001	R^2_{12} = 0.0003	R^2_{12} = 0.0000
	R^2_{23} = 0.0001	R^2_{23} = 0.0000	R^2_{23} = 0.0001	R^2_{23} = 0.0001	R^2_{23} = 0.0001
EDANX40	R^2_{12} = 0.0000	R^2_{12} = 0.0000	R^2_{12} = 0.0000	R^2_{12} = 0.0000	R^2_{12} = 0.0001
	R^2_{23} = 0.0000	R^2_{23} = 0.0000	R^2_{23} = 0.0000	R^2_{23} = 0.0000	R^2_{23} = 0.0000
EDANX41	R^2_{12} = 0.0000	R^2_{12} = 0.0001	R^2_{12} = 0.0001	R^2_{12} = 0.0000	R^2_{12} = 0.0000
	R^2_{23} = 0.0000	R^2_{23} = 0.0000	R^2_{23} = 0.0000	R^2_{23} = 0.0000	R^2_{23} = 0.0000
EDANX53	R^2_{12} = 0.0000	R^2_{12} = 0.0000	R^2_{12} = 0.0000	R^2_{12} = 0.0000	R^2_{12} = 0.0000
	R^2_{23} = 0.0000	R^2_{23} = 0.0000	R^2_{23} = 0.0000	R^2_{23} = 0.0000	R^2_{23} = 0.0000
EDDEP04	R^2_{12} = 0.0000	R^2_{12} = 0.0000	R^2_{12} = 0.0001	R^2_{12} = 0.0000	R^2_{12} = 0.0002
	R^2_{23} = 0.0000	R^2_{23} = 0.0000	R^2_{23} = 0.0000	R^2_{23} = 0.0000	R^2_{23} = 0.0000
EDDEP06	R^2_{12} = 0.0005	R^2_{12} = 0.0004	R^2_{12} = 0.0003	R^2_{12} = 0.0001	R^2_{12} = 0.0008
	R^2_{23} = 0.0000	R^2_{23} = 0.0000	R^2_{23} = 0.0001	R^2_{23} = 0.0000	R^2_{23} = 0.0000
EDDEP29	R^2_{12} = 0.0002	R^2_{12} = 0.0001	R^2_{12} = 0.0000	R^2_{12} = 0.0000	R^2_{12} = 0.0000
	R^2_{23} = 0.0000	R^2_{23} = 0.0000	R^2_{23} = 0.0001	R^2_{23} = 0.0000	R^2_{23} = 0.0000
EDDEP41	R^2_{12} = 0.0001	R^2_{12} = 0.0001	R^2_{12} = 0.0000	R^2_{12} = 0.0000	R^2_{12} = 0.0001
	R^2_{23} = 0.0000	R^2_{23} = 0.0000	R^2_{23} = 0.0000	R^2_{23} = 0.0000	R^2_{23} = 0.0000
HI7	R^2_{12} = 0.0000	R^2_{12} = 0.0000	R^2_{12} = 0.0000	R^2_{12} = 0.0001	R^2_{12} = 0.0001
	R^2_{23} = 0.0000	R^2_{23} = 0.0000	R^2_{23} = 0.0000	R^2_{23} = 0.0001	R^2_{23} = 0.0000
AN3	R^2_{12} = 0.0002	R^2_{12} = 0.0002	R^2_{12} = 0.0001	R^2_{12} = 0.0000	R^2_{12} = 0.0003
	R^2_{23} = 0.0004	R^2_{23} = 0.0002	R^2_{23} = 0.0001	R^2_{23} = 0.0004	R^2_{23} = 0.0003
FATEXP41	R^2_{12} = 0.0001	R^2_{12} = 0.0003	R^2_{12} = 0.0001	R^2_{12} = 0.0001	R^2_{12} = 0.0001
	R^2_{23} = 0.0000	R^2_{23} = 0.0001	R^2_{23} = 0.0001	R^2_{23} = 0.0002	R^2_{23} = 0.0000
FATEXP40	R^2_{12} = 0.0001	R^2_{12} = 0.0001	R^2_{12} = 0.0001	R^2_{12} = 0.0002	R^2_{12} = 0.0000

Measurement properties of the Dutch PROMIS-29 v2.1 profile in people with and without chronic conditions

Ellen BM Elsman, Leo D Roorda, Nynke Smidt, Henrica CW de Vet, Caroline B Terwee

	R^2_{23} = 0.0000	R^2_{23} = 0.0001	R^2_{23} = 0.0001	R^2_{23} = 0.0000	R^2_{23} = 0.0001
Sleep109	R^2_{12} = 0.0004	R^2_{12} = 0.0004	R^2_{12} = 0.0003	R^2_{12} = 0.0005	R^2_{12} = 0.0007
	R^2_{23} = 0.0000	R^2_{23} = 0.0000	R^2_{23} = 0.0001	R^2_{23} = 0.0000	R^2_{23} = 0.0002
Sleep116	R^2_{12} = 0.0002	R^2_{12} = 0.0000	R^2_{12} = 0.0000	R^2_{12} = 0.0003	R^2_{12} = 0.0000
	R^2_{23} = 0.0001	R^2_{23} = 0.0001	R^2_{23} = 0.0000	R^2_{23} = 0.0000	R^2_{23} = 0.0000
Sleep20	R^2_{12} = 0.0004	R^2_{12} = 0.0001	R^2_{12} = 0.0001	R^2_{12} = 0.0005	R^2_{12} = 0.0004
	R^2_{23} = 0.0001	R^2_{23} = 0.0000	R^2_{23} = 0.0000	R^2_{23} = 0.0001	R^2_{23} = 0.0001
Sleep44	R^2_{12} = 0.0001	R^2_{12} = 0.0001	R^2_{12} = 0.0001	R^2_{12} = 0.0001	R^2_{12} = 0.0001
	R^2_{23} = 0.0001	R^2_{23} = 0.0001	R^2_{23} = 0.0001	R^2_{23} = 0.0002	R^2_{23} = 0.0001
SRPPER11_CaPS	R^2_{12} = 0.0000	R^2_{12} = 0.0001	R^2_{12} = 0.0000	R^2_{12} = 0.0000	R^2_{12} = 0.0002
	R^2_{23} = 0.0000	R^2_{23} = 0.0000	R^2_{23} = 0.0000	R^2_{23} = 0.0000	R^2_{23} = 0.0001
SRPPER18_CaPS	R^2_{12} = 0.0000	R^2_{12} = 0.0000	R^2_{12} = 0.0000	R^2_{12} = 0.0000	R^2_{12} = 0.0003
	R^2_{23} = 0.0000	R^2_{23} = 0.0000	R^2_{23} = 0.0000	R^2_{23} = 0.0001	R^2_{23} = 0.0000
SRPPER23_CaPS	R^2_{12} = 0.0003	R^2_{12} = 0.0006	R^2_{12} = 0.0003	R^2_{12} = 0.0002	R^2_{12} = 0.0025
	R^2_{23} = 0.0002	R^2_{23} = 0.0001	R^2_{23} = 0.0001	R^2_{23} = 0.0002	R^2_{23} = 0.0000
SRPPER46_CaPS	R^2_{12} = 0.0000	R^2_{12} = 0.0000	R^2_{12} = 0.0001	R^2_{12} = 0.0000	R^2_{12} = 0.0001
	R^2_{23} = 0.0000	R^2_{23} = 0.0000	R^2_{23} = 0.0001	R^2_{23} = 0.0000	R^2_{23} = 0.0000
PAININ9	R^2_{12} = 0.0002	R^2_{12} = 0.0002	R^2_{12} = 0.0001	R^2_{12} = 0.0003	R^2_{12} = 0.0001
	R^2_{23} = 0.0001	R^2_{23} = -0.0031	R^2_{23} = -0.0007	R^2_{23} = 0.0003	R^2_{23} = 0.0001
PAININ22	R^2_{12} = 0.0000	R^2_{12} = 0.0000	R^2_{12} = 0.0001	R^2_{12} = 0.0000	R^2_{12} = 0.0002
	R^2_{23} = 0.0000	R^2_{23} = -0.0107	R^2_{23} = -0.0249	R^2_{23} = 0.0000	R^2_{23} = 0.0000
PAININ31	R^2_{12} = 0.0007	R^2_{12} = 0.0007	R^2_{12} = 0.0005	R^2_{12} = 0.0004	R^2_{12} = 0.0004
	R^2_{23} = 0.0000	R^2_{23} = -0.0004	R^2_{23} = -0.0005	R^2_{23} = 0.0000	R^2_{23} = 0.0000
PAININ34	R^2_{12} = 0.0000	R^2_{12} = 0.0000	R^2_{12} = 0.0001	R^2_{12} = 0.0001	R^2_{12} = 0.0002
	R^2_{23} = 0.0000	R^2_{23} = 0.0000	R^2_{23} = -0.0060	R^2_{23} = 0.0000	R^2_{23} = 0.0000

COPD: chronic obstructive pulmonary disease

Measurement properties of the Dutch PROMIS-29 v2.1 profile in people with and without chronic conditions

Ellen BM Elsman, Leo D Roorda, Nynke Smidt, Henrica CW de Vet, Caroline B Terwee

Supplementary file 4. Mean domain T-scores[a] with SD for groups differing in gender, age, and chronic diseases

	Physical function	Ability to participate in social roles and activities	Anxiety	Depression	Fatigue	Sleep disturbance	Pain interference	Pain intensity[b]
**Gender**								
Male	53.8 (6.1)	55.8 (7.8)	46.5 (7.6)	45.0 (6.7)	44.1 (9.0)	45.4 (6.6)	46.9 (7.6)	1.6 (2.1)
Female	52.3 (7.0)	54.7 (8.1)	49.1 (8.4)	46.4 (7.5)	46.2 (9.6)	47.7 (7.0)	48.2 (8.3)	2.0 (2.4)
Mean difference (95%-CI)	1.5 (1.4-1.6)	1.1 (1.0; 1.3)	-2.5 (-2.7; -2.4)	-1.4 (-1.5; -1.3)	-2.1 (-2.3; -2.0)	-2.3 (-2.4; -2.2)	-1.3 (-1.4; -1.1)	-0.5 (-0.5; -0.4)
**Age**								
18-39	54.7 (5.3)	55.2 (8.0)	48.4 (8.3)	45.9 (7.3)	47.6 (9.2)	46.5 (6.4)	45.6 (6.9)	1.4 (2.1)
40-64	53.1 (6.4)	54.9 (8.0)	48.0 (8.2)	46.0 (7.3)	45.5 (9.4)	47.0 (7.1)	47.7 (8.1)	1.9 (2.3)
≥65	51.0 (7.8)	55.8 (7.9)	47.7 (8.0)	45.5 (6.8)	43.3 (9.1)	46.5 (7.1)	48.9 (8.4)	2.1 (2.3)
Mean difference 18-39 vs 40-64 (95%-CI)	1.6 (1.4; 1.8)	0.3 (0.1; 0.6)	0.3 (0.1; 0.5)	-0.1 (-0.3; 0.1)	2.1 (1.9; 2.4)	-0.5 (-0.7; -0.3)	-2.1 (-2.4; -1.9)	-0.5 (-0.5; -0.4)
Mean difference 40-64 vs ≥65 (95%-CI)	2.2 (2.0; 2.3)	-0.9 (-1.0; -0.7)	0.3 (0.2; 0.5)	0.5 (0.3; 0.6)	2.2 (1.9; 2.4)	0.5 (0.3; 0.7)	-1.2 (-1.4; -1.0)	-0.2 (-0.3; -0.2)
**Chronic disease**								
No	54.7 (5.0)	56.2 (7.5)	47.4 (7.9)	45.3 (6.8)	44.0 (8.8)	46.1 (6.6)	45.8 (6.8)	1.4 (1.9)
Yes	50.0 (8.0)	53.4 (8.5)	49.0 (8.6)	46.8 (7.8)	47.4 (10.0)	47.9 (7.3)	50.6 (9.0)	2.6 (2.5)
Mean difference (95%-CI)	4.6 (4.5; 4.8)	2.8 (2.6; 2.9)	-1.6 (-1.8; -1.5)	-1.6 (-1.7; -1.4)	-3.5 (-3.6; -3.3)	-1.8 (-1.9; -1.7)	-4.8 (-5.0; -4.7)	-1.3 (-1.3; -1.3)

[a] T-scores, higher scores represent more anxiety/depression; please note that these are not Dutch reference scores, as the sample was not representative for the general Dutch population

[b] Pain intensity is not a T-score but a 0-10 numeric rating scale

Green: hypothesis confirmed; Red: hypothesis unconfirmed

CI: confidence interval, SD: standard deviation

Measurement properties of the Dutch PROMIS-29 v2.1 profile in people with and without chronic conditions

Ellen BM Elsman, Leo D Roorda, Nynke Smidt, Henrica CW de Vet, Caroline B Terwee

Supplementary file 5. Pearson correlations among PROMIS-29 domains and the pain intensity item

	Pain interference	Physical function	Pain intensity	Ability to participate in social roles and activities	Anxiety	Depression	Fatigue	Sleep disturbance
Pain interference	1.00	-.62	0.83	-.40	0.23	0.25	0.41	0.28
Physical function	-.62	1.00	-.55	0.43	-.22	-.24	-.43	-.25
Pain intensity	0.83	-.55	1.00	-.36	0.24	0.26	0.41	0.31
Ability to participate in social roles and activities	-.40	0.43	-.36	1.00	-.44	-.46	-.58	-.31
Anxiety	0.23	-.22	0.24	-.44	1.00	0.66	0.44	0.35
Depression	0.25	-.24	0.26	-.46	0.66	1.00	0.46	0.35
Fatigue	0.41	-.43	0.41	-.58	0.44	0.46	1.00	0.42
Sleep disturbance	0.28	-.25	0.31	-.31	0.35	0.35	0.42	1.00

Green: hypothesis confirmed; Red: hypothesis unconfirmed
